# Association of the single nucleotide polymorphism C1858T of the *PTPN22* gene with unexplained recurrent pregnancy loss: A case-control study

**DOI:** 10.18502/ijrm.v19i10.9819

**Published:** 2021-11-04

**Authors:** Fateme Khanbarari, Nasrin Ghasemi, Mahmood Vakili, Morteza Samadi

**Affiliations:** ^1^Immunology Department, Faculty of Medicine, Shahid Sadoughi University of Medical Sciences, Yazd, Iran.; ^2^Abortion Research Center, Reproductive Sciences Institute, Shahid Sadoughi University of Medical Sciences, Yazd, Iran.; ^3^Health Monitoring Research Center, School of Medicine, Shahid Sadoughi University of Medical Sciences, Yazd, Iran.; ^4^Reproductive Immunology Research Center, Shahid Sadoughi University of Medical Sciences, Yazd, Iran.; ^5^Research Center for Food Hygiene and Safety, Shahid Sadoughi University of Medical Sciences, Yazd, Iran.

**Keywords:** Recurrent pregnancy loss, PTPN22 protein, Single nucleotide polymorphism.

## Abstract

**Background:**

Lymphoid-tyrosine-phosphatase which is encoded by the protein tyrosine phosphatase non-receptor 22 (*PTPN22*) gene plays a pivotal role in the regulation of immune responses by dephosphorylating several signaling intermediates of immune cells.

**Objective:**

Since a balanced immune response has been shown to be important during pregnancy, the purpose of this research was to compare the frequency of the *PTPN22* C1858T polymorphism in women with unexplained recurrent pregnancy loss (URPL) vs. in a control group for the first time.

**Materials and Methods:**

Genomic DNA from 200 individuals with URPL and 200 individuals without URPL (the control group) at the infertility center in Yazd, Iran was isolated using the salting-out method. The *PTPN22* C1858T polymorphism of the two groups was analyzed using polymerase chain reaction-restriction fragment length polymorphism. Genotype frequencies in the women with URPL and the fertile control group were compared using the Chi-square test.

**Results:**

There were significant differences in the frequency of the *PTPN22* 1858T polymorphism in the URPL individuals vs. the healthy controls, i.e. 32.0% and 21.5%, respectively (p = 0.01).

**Conclusion:**

Our findings suggest that the *PTPN22* 1858T polymorphism could play a role in recurrent pregnancy loss. Therefore, genotyping of the mentioned polymorphism can help clinicians to predict the probable risk of URPL.

## 1. Introduction

Recurrent pregnancy loss (RPL) is defined as three or more pregnancy losses before the 20 ${}^{\mathrm{th}}$ wk of gestation (1, 2), Around 50% of RPL cases do not have any clinical detectable trigger. These abortions are referred to as unexplained recurrent pregnancy loss (URPL). It has been assumed that most of them are associated with immunological abnormalities; thus, in recent years much effort has been invested to understand the immunological mechanisms in pregnancy. It has been concluded that a balanced immune response is necessary for a successful pregnancy (1, 3, 4).

Lymphoid-tyrosine-phosphatase (Lyp) plays a key role in maintaining the hemostasis of the immune system. It regulates signaling by dephosphorylating several signaling intermediates of immune cells such as the lymphocyte-specific protein tyrosine kinase. This phosphatase is encoded by the locus *PTPN22* on the short arm of chromosome 1 (1p13.3-13.1). Recent studies have shown an association between autoimmune diseases and *PTPN22* C1858T which is a single nucleotide change at residue 1858 from C to T that results in the substitution of arginine (R) for tryptophan (W) at position 620 of the Lyp enzyme (R620W). Functional consequences of the *PTPN22* C1858T polymorphism remain unresolved, but most studies have indicated that R620 is a gain of function variant (5, 6).

Increased phosphatase activity leads to over-inhibition of immune cell signaling and increases the stimulation threshold of immune receptors such as the B cell receptor (BCR) and the T cell receptor; as a result defective tolerance leads to autoimmune responses such as autoantibody production (7, 8). Studies have shown that there is a statistical association between the *PTPN22* R620W mutation and autoimmune diseases such as rheumatoid arthritis (9), Type 1 diabetes (10), Graves' disease (11), and systemic lupus (12). This single nucleotide polymorphism (SNP) is a risk factor for autoimmune diseases in which autoantibodies are involved (13). So far, no studies have been conducted to investigate the association between the mentioned polymorphism and URPL, but a statistically significant association has been found between the *PTPN22* R620W polymorphism and endometriosis.

Studies in autoimmune individuals and healthy individuals who carry the mentioned SNP have shown that functional consequences of *PTPN22* C1858T on the immune response can lead to pregnancy loss, due to the prominent role of Lyp in modulating the immune response. Given the importance of a balanced immune response for pregnancy outcomes, the aim of the present study was to explore the association between *PTPN22* C1858T and URPL for the first time.

## 2. Materials and Methods

### Study populations

In this case-control study, 200 women with at least three unexplained consecutive miscarriages before the 20 ${}^{\mathrm{th}}$ wk of gestation with the same partner who were referred to the Yazd Reproductive Sciences Institute in Iran between May 2015 and March 2016 were selected as the case group and 200 healthy, fertile, non-pregnant women with at least one child and no history of abortion were selected as the control group. They were recruited into the study after signing a consent form.

The inclusion criteria for the control group included fertility and having at least one child so women who had had even one miscarriage were excluded from the control group. Case group participants were examined for uterus anomalies, chromosomal abnormalities, hormonal disorders, and infection, as these can be causes of pregnancy loss. After examination, women without any known causes of abortion were chosen as the case group and women with any of the aforementioned conditions were excluded from the study. In addition, the semen of the case group spouses was analyzed. People with known causes of recurrent miscarriage or a history of infertility were excluded.

### Genotyping of the *PTPN22* 1858 polymorphism

Blood samples were collected in tubes containing Ethylenediaminetetraacetic acid. Peripheral blood leukocyte DNA was extracted using the salting-out method. For the determination of the *PTPN22* alleles, polymerase chain reaction followed by restriction fragment length polymorphism assay (PCR-RFLP) was performed.

The amplification reaction was done in 25 μl which contained 3 μl of DNA, 13 μl of master mix (Ampliqon, Denmark), 7 μl of H ${}_{2}$ O, 1 μl of forward primer (5'-TGCCCATCCCACACTTTAT-3') and 1 μl of reverse primer (5'-ACCTCCTGGGTTTGTACCTTA-3'). Thermal cycling was performed with an initial denaturation step at 95°C for five min, then 35 cycles of denaturation at 94°C for one min, annealing temperature 56°C for 45 sec, extension at 72°C for 45 sec, and a final extension at 72°C for five min.

**Figure 1:**
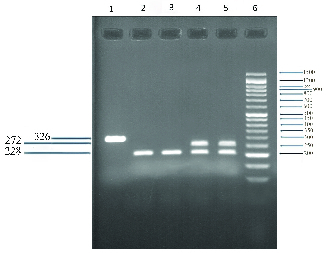
Genotyping of the *PTPN22* 1858 polymorphism by PCR-RFLP. Lane 6: 50bp DNA ladder, Lane 5,4: Samples with heterozygous patterns (CT 272 & 228 bp), Lane 3,4: Samples with homozygote pattern (CC 228 bp), Lane 1: PCR product (326 bp).

The 326-bp PCR product was run on 1% agarose gel (Merk, Germany). The quality was confirmed using green viewer (Parstous, Iran) and a UV transiluminator. Then for RFLP, the PCR product was treated with 1U of Rsa1 enzyme (New England Biolabs, USA) at 37°C for two hr and was subsequently analyzed on 1.5% agarose gel. The 620W variant (T allele) in the presence of restriction enzyme breaks down to a segment of 272 bp, and the C allele (wild type) to a fragment of 228 bp (Figure 1).

### Ethical considerations

The study protocol was approved by the Ethics Committee of Shahid Sadoughi University of Medical Sciences, Yazd, Iran (Code: IR.SSU.MEDICINE.REC.1394.264). Written informed consent was provided by all participants.

### Statistical analysis

The data were analyzed using the Statistical Package for the Social Sciences, version 16 software (SPSS, IBM, Armonk, NY, USA). The Chi-square test was used to compare the frequency of genotypes and alleles between the two groups. Odds ratios were calculated with a confidence interval of 95%. The *t* test was used to compare the mean ages in the URPL and control groups and p < 0.05 was considered statistically significant.

## 3. Results

There was no statistical difference found between the mean age in the case vs. control groups (the mean age in the case and control groups was 35.15 and 34.92, respectively; p = 0.55). None of the participants had a history of infection or illness.

In the case group, 160 (80%) of the participants had pain and bleeding during an abortion, and 40 (20%) had no pain or bleeding. More women aged 35 yr and older had experienced miscarriages than women aged < 35 yr, but this difference was not significant (p = 0.1). The Chi-square test showed that the number of miscarriages was not associated with the polymorphism (Table I).

The *t* test indicated that there was no association between the genotype and the time of miscarriage during pregnancy (the mean of the abortion time in individuals who had *PTPN22* 1858T and individuals who had wild type was 6.03 and 5.76, respectively; p = 0.57).

This study was conducted to determine the association between the *PTPN22* R620W polymorphism and the probability of URPL. The statistical analyses showed a significant difference in the presence of the polymorphism between the URPL and control participants. The frequencies of the T allele in the control and the case groups were 10.75% and 16.00%, respectively (p = 0.02). 32% of case group had the *PTPN22* 620W variant, while the control value was 21.5% (p = 0.01). The allelic and genotypic frequencies of the case and control groups are presented in Table II.

**Table 1:** Genotype frequencies of the *PTPN22 *1858 SNP in URPL individuals, stratified by the number of miscarriages


**Number of miscarriages**	**CT**	**CC**	**p-value**
**3**	46 (35.9)	82 (64.1)	
**4**	15 (27.3)	40 (72.7)	
**5**	3 (17.6)	14 (82.4)	0.21
Data presented as n (%). Chi-square test and p < 0.05 was considered significant			

**Table 2:** Genotypes and allelic frequencies of *PTPN22* 1858 for the case and control groups


**Type**		**Case group**	**Control group**	**OR (95% CI)**	**p-value**
**Genotype **					
	**TT**	0 (0)	0 (0)	
	**CC**	136 (68)	157 (78.5)	
	**CT**	64 (32)	43 (21.5)	1.72 (1.07-2.76)	0.01
**Allele **					
	**C**	336 (84)	357 (89.25)			
	**T**	64 (16)	43 (10.75)	1.58 (1.02-2.44)	0.02
Data presented as n (%). Chi-square test and p < 0.05 was considered significant. OR: Odds ratio, CI: Confidence interval					

## 4. Discussion

This study demonstrated a relationship between the *PTPN22* 1858T polymorphism and susceptibility to URPL; indeed, the frequency of the 1858T polymorphism was significantly higher in the women with URPL compared to in the healthy controls (32.0% vs. 21.5%). This study, like previous studies, indicates the importance of a balanced immune response for a successful pregnancy. Lyp, as the protein tyrosine phosphatase unique to the immune tissues, plays a vital role in maintaining a balanced immune response. The *PTPN22* 620W polymorphism increases Lyp activity, which results in reduced immune cell signaling and the development of autoimmune responses (14).

The BCR signaling threshold plays a crucial role in the fate of the autoreactive B cell. Apoptosis occurs in immature B cells which react to self-antigen with high affinity, whereas defective BCR signaling causes autoreactive B cells to escape from apoptosis and central tolerance.

The *PTPN22* R620W mutation disturbs B cell signaling and increases the stimulation threshold of BCR. An increased stimulation threshold of B cells affects the selection of these cells during tolerance, causes resistance to BCR-derived apoptosis and survival of autoreactive cells, and ultimately leads to loss of tolerance, resulting in increased autoreactive progenitor cells in the peripheral blood. Individuals carrying the 1858T allele deliver a source of autoreactive B cells that can produce autoantibodies. Previous studies have shown that in people carrying the 1858T allele, the abundance of anergic IgD ${}^{+}$ IgM ${}^{-}$ B cells is higher. Recent studies have indicated that these B cells may be the peripheral source of autoreactive cells (15-17).

In addition, impaired Lyp enzymes cause changes in thymic selection. Although there has been no report on the role and involvement of Lyp in the positive and negative selection of the thymus, the high expression of Lyp in thymocytes indicates its major role in the T cell education process in the thymus. In fact, the 620W Lyp causes autoreactive cells to escape from the negative selection mechanism of the thymus and leads to loss of central T cell tolerance through over-inhibition of T cells.

Furthermore, the mentioned mutation reduces the efficiency of Treg activities and ultimately degrades the peripheral tolerance by increasing the signaling threshold of the Treg (8, 18). Finally, central and peripheral tolerance failure of immune cells results in self-immune responses in the body. Thus, the *PTPN22* 1858T polymorphism is considered as one of the prominent risk factors for autoimmune diseases outside the Human leukocyte antigen locus (14).

A meta-analysis showed that there was a statistically significant correlation between the presence of the 1858T allele and autoimmune diseases such as rheumatoid arthritis, systemic lupus erythematosus, Graves' disease, Type 1 diabetes, and juvenile idiopathic arthritis (19, 20). Autoimmune reactions increase the incidence of recurrent abortions (21). For example, the rate of recurrent miscarriage is higher in women with systemic lupus erythematosus than in healthy women; in point of fact anti-nuclear antibody and anti-cardiolipin antibody autoantibodies seemed to be important risk factors for the incidence of RPL (22, 23) and previous studies have indicated that there is a statistically significant correlation between the *PTPN22* R620W mutation and the presence of these two autoantibodies (12, 24). It can be deduced that the *PTPN22* 620W polymorphism could increase the likelihood of recurrent abortions by increasing autoantibody production.

So far, no study has been conducted to examine the association between this SNP and URPL. However, few studies have been performed to investigate the association between the *PTPN22* 620W polymorphism and endometriosis, as one of the common complications of fertility. For example, a study on the Italian population showed that the frequency of the *PTPN22* 620W allele in individuals suffering from endometriosis was higher than in healthy subjects. A study on the Brazilian population in 2009 also showed that there was a statistically significant correlation between the presence of the *PTPN22* 620W polymorphism and endometriosis (25, 26).

In addition, the correlation between this variant and abortion can be discussed from another perspective. Cytokines are divided into two types (pro-inflammatory and anti-inflammatory), which are produced by Th1 and Th2 cells, respectively. During pregnancy, TCD4+ cells are more likely to differentiate into Th2 cells and produce anti-inflammatory cytokines such as IL-4 and IL-10, while pro-inflammatory cytokines such as TNF-α and IFN-γ are harmful to pregnancy (27). The *PTPN22* 1858T allele alters the balance of Th1/Th2 cytokine profiles. Studies have shown that individuals who carry *PTPN22* 1858T exhibit more pro-inflammatory cytokines such as IFN-γ and fewer Th2 cytokines (IL-4 and IL-10) than individuals homozygous for the C allele. Therefore, it can be stated that the *PTPN22* 620W mutation may lead to URPL by skewing of cytokine profiles toward Th1 (28, 29).

Based on our findings, it can be concluded that functional consequences of *PTPN22* C1858T on immune responses can lead to pregnancy loss. To the best of our knowledge, no other study has examined the association between this polymorphism and URPL so far. This study compared the frequency of the *PTPN22* 620W polymorphism between women with recurrent pregnancy loss and healthy people. Based on the results of this study, the *PTPN22* 1858T polymorphism seems to influence the incidence of URPL. Our team also conducted a study to ascertain the association between URPL and the rs2488457 polymorphism, which is located in the promoter region of *PTPN22*: the frequency of the rs2488457 polymorphism was higher in URPL individuals compared with in fertile controls (7.00% vs. 4.75%); however, there was no significant difference (p = 0.16) (30). These two types of polymorphisms are completely independent in terms of inheritance pattern and function. *PTPN22* 1858T is more common in European populations, especially of the Caucasian race, while the rs2488457 polymorphism is more prevalent in East Asia. Indeed, different ethnic backgrounds may have caused this discrepancy in the results. Our findings suggest that the frequency distribution of the mentioned polymorphisms in the Iranian population was similar to in European populations, but further epidemiologic research should be performed to confirm our findings.

Given that the Yazd Infertility Center is located in the center of Iran and given that people are referred to this center from different parts of the country, the results can be generalized to the entire population of the country. Therefore, the obtained information from this study can be useful in understanding the genetic structural information of the Iranian population.

## 5. Conclusion

We can conclude that *PTPN22* 1858T could be considered as one of the causes of URPL and targeting of this functional variant can help to diagnose women at risk of recurrent pregnancy loss. Therefore, the results of this study can be helpful in explaining and clarifying one of the possible causes of URPL and for diagnostic purposes.

##  Conflict of Interest

The authors declare that there is no conflict of interest.
